# Prevalence and Antimicrobial Susceptibility of *Escherichia coli* O157:H7 Isolated From Slaughtered Sheep, Goats, and the Surrounding Environment at Haramaya Municipal Abattoir, Eastern Ethiopia

**DOI:** 10.1155/vmi/5525883

**Published:** 2026-04-07

**Authors:** Aklilu Asmelash, Yusuf Abrahim, Ashenafi Syoum, Gebremedhin Gebrezgabiher

**Affiliations:** ^1^ Department of Veterinary Medicine, College of Veterinary Medicine and Animal Sciences, Samara University, P. O. Box 132, Samara, Ethiopia, su.edu.et

**Keywords:** abattoir, antibiogram, carcass, *Escherichia coli* O157:H7, Ethiopia, goat, Haramaya, prevalence, sheep

## Abstract

Shiga toxin–producing *Escherichia coli* O157:H7 (STEC) is a major foodborne pathogen associated with illnesses such as diarrhea, hemorrhagic colitis, and hemolytic–uremic syndrome. Due to the limited data regarding the prevalence of *E. coli* O157:H7 in the study area, this cross‐sectional study was conducted at the Haramaya municipal abattoir in Ethiopia from February to August 2021 to evaluate the prevalence and antibiotic resistance profile of the pathogen in slaughtered sheep, goats, and the surrounding environment. Systematic random sampling was used to obtain 440 samples from 78 sheep and 32 goats. These samples consisted of swabs from the external and internal surfaces of carcasses, skin swabs, and fecal samples. Additionally, 24 environmental samples, comprising 12 separate hand and knife swabs, were purposefully collected. All the samples were enriched in modified tryptone broth containing novobiocin and then streaked onto EMB agar for preliminary *E. coli* detection. Colonies confirmed as *E. coli* through biochemical tests were further cultured on sorbitol MacConkey agar, and non‐sorbitol‐fermenting isolates were subjected to latex agglutination for serogroup identification. Susceptibility testing was performed on all *E. coli* O157:H7 isolates against eight selected antibiotics. Only 13 of the total 464 samples analyzed (2.8%) were positive, with most originating from sheep (69.2%, 9/13), followed by goats (23.1%, 3/13) and one from a knife (7.7%, 1/13). The prevalence results indicate that slaughtered sheep and goats are significant reservoirs of *E. coli* O157:H7, with the detection in environmental samples highlighting critical gaps in abattoir hygiene that facilitate carcass contamination. The isolates were fully susceptible to gentamicin and kanamycin but resistant to ampicillin, erythromycin, amoxicillin, and vancomycin, and all exhibited multidrug resistance. The presence of *E. coli* O157:H7 and antibiotic‐resistant isolates signals a public health risk for meat handlers and consumers, reinforcing the necessity of applying proper control measures in abattoirs and during meat consumption.

## 1. Introduction

Foodborne pathogens are among the leading causes of human illness and death worldwide, with many being zoonotic and of food‐animal origin [1–3]. Animal‐derived foods such as milk, meat, eggs, and fish are high risk due to their susceptibility to microbial contamination and growth [4], posing significant health risks when consumed raw and/or undercooked [5, 6].

Several harmful bacteria linked to animal‐origin foods affect both humans and animals, notably *Escherichia coli* (*E. coli* O157:H7), *Staphylococcus aureus*, *Salmonella*, *Campylobacter*, and *Listeria monocytogenes* [7, 8].

Six major *E. coli* pathotypes, namely, Shiga toxin‐producing *E. coli* (STEC), enteropathogenic *E. coli* (EPEC), enterotoxigenic *E. coli* (ETEC), enteroinvasive *E. coli* (EIEC), enteroaggregative *E. coli* (EAEC), and diffusely adherent *E. coli* (DAEC), are known to cause gastrointestinal disorders in humans [9]. Their classification is based on the particular virulence traits that characterize each group. For STEC, the main virulence trait is toxin production, which inhibits protein synthesis and is encoded by the stx1 and stx2 genes. Other factors, such as intimin (encoded by the eae gene) and autoagglutinating adhesins, may also be involved [10]. Notably, *E. coli* O157:H7 (a STEC strain) is nonpathogenic to ruminants but can cause severe diseases in humans, including diarrhea, hemorrhagic colitis, hemolytic‐uremic syndrome, and sometimes death [11].

*E. coli* O157:H7 inhabits the gastrointestinal tracts of humans and animals and is transmitted primarily through contaminated raw or undercooked meat. Vulnerable populations include infants, children, pregnant women, and immunocompromised individuals [12–14]. The World Health Organization (WHO) has recognized *E. coli* O157:H7 as a major antibiotic‐resistant pathogen among 12 bacterial families, posing significant health risks to humans and animals [15].

Beyond its widespread distribution, the increasing emergence and dissemination of antibiotic‐resistant bacteria, especially multidrug‐resistant (MDR) zoonotic foodborne pathogens, has become a major global issue [16, 17]. These antimicrobial‐resistant organisms can reach humans via the food chain and originate from food‐producing animals [18]. Studies from different regions of Ethiopia have shown a notable increase in the resistance of *E. coli* O157:H7 to frequently used antibiotics [19, 20].

In Ethiopia, slaughtering is often conducted in unhygienic environments, putting meat safety at risk [21]. Additionally, the lack of proper monitoring for foodborne pathogens, combined with limited education and training for slaughterhouses and butcher staff and inadequate hygiene practices by food handlers, greatly contributes to the heightened risk of contamination with pathogens such as EHEC [1]. Meat consumption in Ethiopia favors beef, mutton, and chevon, with raw meat consumption being common, increasing the potential for foodborne illness if hygiene and temperature controls are inadequate [22, 23].

Surveillance of foodborne pathogens is crucial for managing and preventing risks throughout food production, processing, and distribution [24]. Despite this, comprehensive data on foodborne infections in low‐income countries, where the impact of such diseases is considerably greater than that in high‐income countries, are lacking [25]. Although some data exist regarding *E. coli* O157:H7 in the surrounding area, specifically at the Dire Dawa municipal abattoir, reports of its presence and antimicrobial resistance patterns at the Haramaya municipal abattoir remain limited. Therefore, this study was conducted to evaluate the prevalence and antibiotic resistance profile of *E. coli* O157:H7 in sheep and goats slaughtered at this site.

## 2. Materials and Methods

### 2.1. Description of the Study Area

**FIGURE 1:**
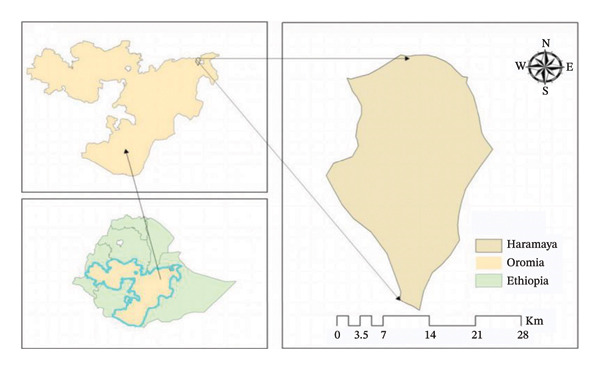
Map of the study area.

The study was carried out at the Haramaya municipal abattoir, located in Haramaya town (Figure 1) within the East Hararghe administrative zone of the Oromia Region, Eastern Ethiopia, approximately 510 km from Addis Ababa. The study site lies at a latitude of 9°24′N and a longitude of 42°01′E, with an elevation ranging from 1600 to 2100 m above sea level [26, 27].

Mixed crop–livestock farming is the dominant production system in rural areas. The primary livestock species reared include cattle, sheep, goats, camels, donkeys, and poultry. In Haramaya woreda, the livestock population consists of approximately 120,145 goats and 69,950 sheep [26, 27].

### 2.2. Study Population

The study involved apparently healthy sheep and goats slaughtered at the Haramaya municipal abattoir during the study period. On average, approximately 170 animals were slaughtered each day. These animals were sourced primarily from nearby markets, including Dawe, Kersa, and Haramaya.

### 2.3. Study Design and Sampling Method

A cross‐sectional study was conducted from February to August 2021. Individual animals were selected via a systematic random sampling method on the basis of their identification codes prior to slaughter.

### 2.4. Sample Size Determination

The minimum required sample size for this study was determined via the formula provided by Thrusfield [28]. The calculation was based on an expected prevalence of 2.15% for goats [29] and 5.4% for sheep [30], resulting in sample sizes of 32 goats and 78 sheep. In addition, 24 environmental samples, including 12 hand swabs and 12 knife swabs, were purposefully collected. A total of 464 samples were collected and analyzed.

(1) n=Z2Pexp1−Pexpd2.

where

• *n* = sample size,
• *d* ^2^ = absolute precision of 0.05,
• Pexp = expected prevalence, and
• Z = statistic for a level of confidence of 1.96.

### 2.5. Study Samples

A total of 464 samples were collected for the study. This included 128 samples from goats, with 32 samples each from four categories: carcass outside swabs, carcass inside swabs, skin swabs, and cecal contents. From sheep, 312 samples were collected, with 78 for each of the same four sample types. In addition, 24 environmental samples were obtained, consisting of swabs from slaughterhouse workers’ hands and knives.

### 2.6. Procedures for Sample Collection

Prior to sample collection, each study animal was identified via its designated identification code. Carcass swabs, skin swabs, fecal samples, hand swabs, and knife swabs were then collected separately, following the identification codes. To avoid cross‐contamination, samples were taken at different collection facilities.

The external surfaces of the carcasses were sampled via swabs taken from the rump, midline, and brisket areas just before chilling, following the methods described by McEvoy et al. [31]. Sterile cotton swabs moistened with approximately 10 mL of buffered peptone water (Oxoid Ltd., Hampshire, England) were used to swab the surfaces, which were then moved horizontally and then vertically several times across each area. For internal surface sampling, areas unlikely to come into contact with other carcasses, swabs were collected from the thoracic and pelvic regions on both sides through the evisceration opening via the same technique. Disposable sterile gloves were worn and changed after each animal was sampled to prevent cross‐contamination.

Skin swab samples were collected using the procedure described by Abreham et al. [3] with sterile cotton swabs (2 × 3 cm) presoaked in approximately 10 mL of buffered peptone water (Oxoid Ltd., Hampshire, England). Swabbing was carried out on the animals’ neck region near the bleeding site, covering an area of approximately 10 × 10 cm just before slaughter. In addition, the ventral midline skin was swabbed at the midline to evaluate potential contact with the carcass during the flaying process. After collection, the swab shafts were broken by pressing them against the inner wall of the test tube and then discarded.

Approximately 10 g of cecal content was collected aseptically from slaughtered sheep and goats after evisceration, following the method described by Dulo et al. [32]. The fecal material was gently compressed, and the resulting mixture was transferred into a sterile universal bottle. All the samples were labeled and transported on ice in a cooler box to the laboratory, where they were stored at 2°C–3°C overnight and processed the next day.

The environmental samples were collected via the methods outlined by Dulo et al. [32]. Swabs were taken from both hands of the abattoir workers, as well as from the blades and handles of the slaughter knives.

### 2.7. Sample Preparation and Selective Enrichment

Fecal samples (10 g) were carefully weighed and transferred into sterile stomacher bags, after which modified tryptone soy broth (mTSB) supplemented with 20 mg/L novobiocin (mTSB + *n*) was added at a 1:9 sample‐to‐broth ratio. The contents were then blended using a stomacher (Seward Stomacher 400, Seward, London, UK) at low speed for 30 s. For the remaining swab samples, 90 mL of mTSB + *n* was added, and the samples were mixed thoroughly via a vortex mixer [3].

### 2.8. Culture and Isolation of *E. coli* O157:H7

**FIGURE 2:**
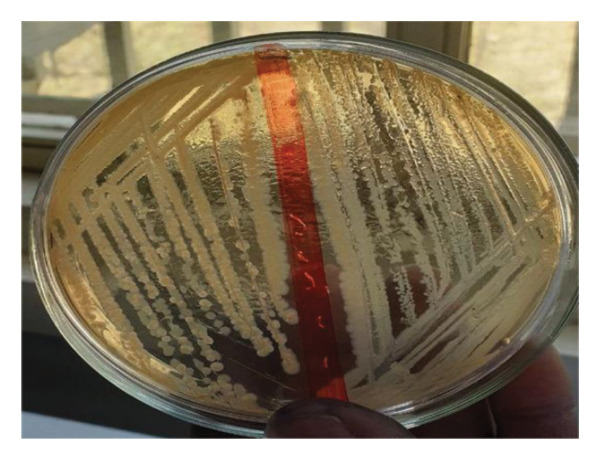
Growth of *E. coli* on SMAC agar plates.

All enriched fecal and swab samples were subsequently streaked onto eosin methylene blue (EMB) agar (Difco Laboratories, USA) for preliminary *E. coli* detection and incubated aerobically at 37°C for 24 h. Colonies exhibiting characteristic *E. coli* morphology, a greenish metallic sheen with dark purple centers, were identified via the API 20E gallery (API20 E/20100, bioMerieux, Marcy l’Etoile, France) [33] and then subjected to additional testing for *E. coli* O157:H7. Briefly, two to three biochemically confirmed *E. coli* strains were subcultured onto sorbitol MacConkey agar (Oxoid) supplemented with 0.05 mg/L cefixime and 2.5 mg/L tellurite (SMAC‐CT) and incubated at 37°C for 18–24 h. After incubation, the SMAC‐CT agar plates were examined for non‐sorbitol‐fermented colonies, which appeared slightly transparent, nearly colorless with a weak pale brownish appearance, and approximately 1 mm in diameter [34, 35] (Figure 2). As a positive control, the *E. coli* O157:H7‐type strain (ATCC 43895) was cultured in 10 mL of brain heart infusion (BHI) broth (Oxoid) at 37°C for 24 h, then streaked onto SMAC‐CT agar and incubated again for 24 h at 37°C [33].

### 2.9. Serological Identification of *E. coli* O157:H7

All the isolates that did not ferment sorbitol on SMAC‐CT agar (Figure 2) were further analyzed through slide agglutination via the Oxoid *E. coli* O157 latex test kit [33, 36].

### 2.10. Antimicrobial Susceptibility Test and Determination of Resistance to Multiple Antibiotics

All 13 *E. coli* O157:H7 isolates were evaluated for antimicrobial susceptibility in vitro via the agar disk diffusion technique following the procedure described by Bauer et al. [37]. Eight antibiotic disks from six different classes (Mast Group Ltd., Merseyside, U.K.) were used: penicillins (amoxicillin 10 μg, ampicillin 25 μg), macrolides (erythromycin 15 μg), aminoglycosides (gentamicin 10 μg, kanamycin 30 μg), chloramphenicol (30 μg), tetracycline (30 μg), and glycopeptides (vancomycin 30 μg). These antibiotics were selected on the basis of their availability and frequent use in both veterinary and human medicine within the study area.

Pure *E. coli* cultures were incubated in Trypticase Soy Broth (Oxoid Limited, Basingstoke, UK) at 37°C for 18 h. The culture turbidity was standardized to a 0.5 McFarland level via sterile Trypticase Soy Broth before being spread onto Müller Hinton agar (Oxoid, Basingstoke, UK). Antibiotic disks were then applied to the agar surface, and the plates were incubated at 37°C for 24 h. The diameters of the inhibition zones were recorded, and interpretations were made following the Clinical and Laboratory Standards Institute (CLSI) guidelines [38] (Table 1). Isolates that were resistant to one or more antibiotics in three or more different classes were considered MDR [39]. The multiple antibiotic resistance (MAR) index was determined via the method described by Krumperman [40] and was calculated as a/b, where “a” represents the number of antibiotics to which the isolate was resistant and “b” represents the total number of antibiotics tested.

**TABLE 1:** Antibiotic discs used to test *E. coli* O157:H7 and their respective concentrations.

**Antibiotic discs**	**Concentration**	**Diameter of zone of inhibition in millimeter (mm)**		
		**Resistant**	**Intermediate**	**Susceptible**
		**≤**		**≥**

Ampicillin (Amp)	10 μg	13	14–16	17
Amoxicillin (Aml)	10 μg	13	14–17	18
Erythromycin (E)	15 μg	13	14–22	23
Chloramphenicol (C)	30 μg	12	13–17	18
Gentamicin (Cn)	10 μg	12	13–14	15
Kanamycin (K)	30 μg	13	14–17	18
Vancomycin (Va)	30 μg	14	15–16	17
Tetracycline (Te)	30 μg	11	12–14	15

### 2.11. Data Analysis

All raw data gathered during the study were compiled, entered, and coded via Microsoft Excel 2007 and subsequently imported into SPSS version 20.0 (Statistical Package for the Social Sciences) for analysis. Prevalence rates, both overall and by sample type, were calculated by dividing the number of positive samples by the total number of samples examined. Descriptive statistics, including frequencies and percentages, were also computed.

## 3. Results

### 3.1. Overall Prevalence of *E. coli* O157:H7 Isolates

**FIGURE 3:**
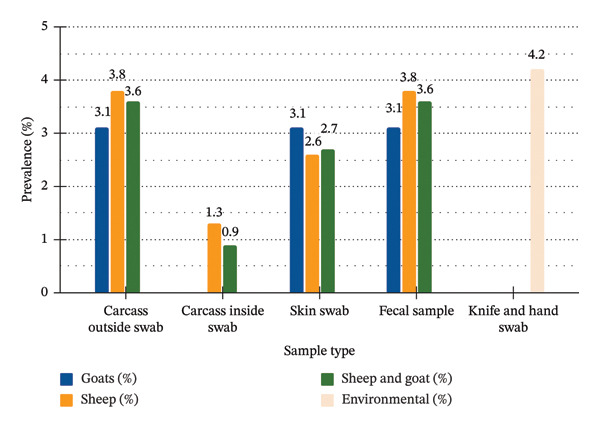
Prevalence of *E. coli* O157:H7 by sample type and species of animals examined.

Among the 464 samples analyzed, 85 (18.32%) were contaminated with *E. coli*, whereas only 13 samples (2.80%) tested positive for *E. coli* O157:H7, as depicted in Table 2 and Figure 3. The *E. coli* O157:H7 strains were detected in fecal samples, skin swabs, carcass surface swabs (both outside and inside), and knives used at the abattoir.

**TABLE 2:** Prevalence of *E. coli* O157:H7 by sample type and species of animals examined.

Sample type	Sources					
	Goat		Sheep		Sheep and goat	
	No examined	Positive *n* (%)	Total	Positive *n*(%)	Total	Positive *n*(%)
Carcass outside	32	1 (3.1)	78	3 (3.8)	110	4 (3.6)
Carcass inside	32	0 (0)	78	1 (1.3)	110	1 (0.9)
Skin swab	32	1 (3.1)	78	2 (2.6)	110	3 (2.7)
Feces	32	1 (3.1)	78	3 (3.8)	110	4 (3.6)
Knife and hand swabs	—		—		24	1 (4.2)
Total	128	3 (2.34)	312	9 (2.88)	464	13 (2.8)

Among the 13 positive samples, 69.2% (9/13) originated from sheep, 23.1% (3/13) from goats, and 7.7% (1/13) from a knife. In goats, *E. coli* O157:H7 was isolated from carcass outside swabs, skin swabs, and fecal samples, each with a prevalence of 3.1%. It was not detected in the carcasses inside swabs. In sheep, the bacteria were identified in 3.8% of the carcass outside swabs, 1.3% of the carcass inside swabs, 2.6% of the skin swabs, and 3.8% of the fecal samples.

Owing to the low number of positive cases and limited sample size, conducting separate statistical analyses for sheep and goats was challenging. For prevalence determination, an animal was considered positive for *E. coli* O157:H7 only if the pathogen was detected in its fecal sample. Contamination found on carcass surfaces or skin was treated as an indicator of external contamination rather than infection. On the basis of these criteria, only 4 out of the 110 animals tested were considered truly positive for *E. coli* O157:H7.

### 3.2. Antibiogram Profiling

#### 3.2.1. Overall Antimicrobial Susceptibility Test

In vitro antimicrobial susceptibility testing of the 13 *E. coli* O157:H7 isolates against eight selected antibiotics revealed that all the isolates (100%) were susceptible to gentamicin and kanamycin. In contrast, complete resistance (100%) was observed against ampicillin, erythromycin, amoxicillin, and vancomycin. Furthermore, a high level of resistance was recorded against chloramphenicol, with 92.3% of the isolates showing resistance (Table 3).

**TABLE 3:** Antimicrobial susceptibility test results for the *E. coli* O157:H7 isolates.

Antimicrobial agent	Susceptibility and resistance pattern of all *E. coli* O157:H7 isolates			
	Disc concentration	S (%)	R (%)	I (%)
Te	30 µg	0	69.23	30.7
Amp	10 μg	0	100	0
E	15 μg	0	100	0
Cn	10 μg	100	0	0
Aml	10 μg	0	100	0
C	30 μg	0	92.3	7.7
Va	30 µg	0	100	0
K	30 μg	100	0	0

*Note:* Keywords: %: Percent, S: sensitive, I: intermediate, R: resistant, Te: tetracycline, AMP: ampicillin, E: erythromycin, CN: gentamicin, Aml: amoxicillin, C: chloramphenicol, Va: vancomycin, K: kanamycin.

Table 4 presents the antimicrobial resistance patterns and MAR indices of the *E. coli* O157:H7 isolates obtained from various sample types. The MAR indices ranged from 0.63, indicating resistance to five antibiotics, to 0.75, corresponding to resistance to six antibiotics.

**TABLE 4:** Antibiotic resistance profiles and multiple antibiotic resistance indices of individual *E. coli* O157:H7 strains isolated from different sheep and goat samples.

No.	Source of sample	Resistance pattern	Resistance to how many antimicrobials	MAR index
1	Sheep feces	AmpEAml CVa	5	0.63
2	Sheep carcass inside swab	TeAmpEAmlCVa	6	0.75
3	Sheep carcass outside swab	TeAmpEAmlVa	5	0.63
4	Sheep carcass outside swab	AmpEAmlCVa	5	0.63
5	Sheep carcass outside swab	TeAmpEAmlCVa	6	0.75
6	Sheep skin swab	AmpEAmlCVa	5	0.63
7	Sheep skin swab	TeAmpEAmlCVa	6	0.75
8	Sheep feces	TeAmpEAmlCVa	6	0.75
9	Sheep feces	TeAmpEAmlCVa	6	0.75
10	Goat carcass outside swab	AmpEAmlCVa	5	0.63
11	Knife	TeAmpEAmlCVa	6	0.63
12	Goat skin swab	TeAmpEAmlCVa	6	0.63
13	Goat feces	TeAmpEAmlCVa	6	0.63

*Note:* AmpEAmlCVa: ampicillin–erythromycin–amoxicillin–chloramphenicol–vancomycin; TeAmpEAmlCVa: ampicillin–erythromycin–amoxicillin–chloramphenicol–vancomycin; TeAmpEAmlVa: tetracycline–ampicillin–erythromycin–amoxicillin–vancomycin.

Among the 13 isolates, 38.5% were resistant to five antimicrobial agents, whereas the remaining 61.5% were resistant to six antimicrobial agents. In total, three distinct resistance patterns were identified. The most frequent pattern, TeAmpEAmlCVa (tetracycline–ampicillin–erythromycin–amoxicillin–chloramphenicol–vancomycin), was found in eight isolates. The second most common pattern, AmpEAmlCVa (ampicillin–erythromycin–amoxicillin–chloramphenicol–vancomycin), was observed in four isolates. One isolate exhibited the pattern TeAmpEAmlVa (tetracycline–ampicillin–erythromycin–amoxicillin–vancomycin).

All *E. coli* O157:H7 isolates (100%) were classified as MDR. Notably, highly related resistance patterns were observed in isolates from different sources.

## 4. Discussion

This study provides the first comprehensive assessment of *E. coli* O157:H7 at the Haramaya municipal abattoir in Eastern Ethiopia. While cattle are often cited as the primary reservoir [41], the findings of this study indicate that sheep and goats are equally significant in the pathogen transmission. These findings agree with results from Mojo, Ethiopia [30]. The overall prevalence of *E. coli* O157:H7 in sheep and goats was 3.6%, which is consistent with the findings of Rahimi et al. [42], who reported a prevalence of 3.27% in Fars and Khuzestan Provinces, Iran. Lower prevalence rates have been reported in other regions, such as the 1.33% reported by Asim et al. [43] in Islamabad, Pakistan, and the 1% reported by Al‐Ajimi et al. [44] in Al Ain, United Arab Emirates. Conversely, a higher prevalence was reported by Abreham et al. [3], who reported a rate of 8.3% in sheep and goats at an export abattoir in Modjo, Ethiopia. One possible reason for this discrepancy is that the present study did not include intestinal mucosal swabs, sample types that, in the previous study, yielded the greatest number of isolates.

The pathogen was detected in both species, with a prevalence of 3.8% in sheep and 3.1% in goats. In goats, the pathogen was detected in carcass outside swabs (3.1%), skin swabs (3.1%), and fecal samples (3.1%). In sheep, the detection rates were 3.8% for carcass outside swabs, 1.3% for carcass inside swabs, 2.6% for skin swabs, and 3.8% for fecal samples. This suggests that carcass contamination occurs primarily through contact with fecal matter or contaminated skin during slaughter and processing [33].

The prevalence of *E. coli* O157:H7 on the exterior surface of carcasses was 3.1% in goats and 3.8% in sheep. These results are consistent with earlier studies by Dulo et al. [32], who reported a prevalence of 3.2% in goats from the Somali Region of Ethiopia, and Abreham et al. [3], who reported a 2.5% prevalence in sheep at an export abattoir in Modjo, Ethiopia. In contrast, higher prevalence rates have been reported by Bekele et al. [45], who reported a 9.4% prevalence in sheep and a 7.8% prevalence in goats in Addis Ababa, and by Aman et al. [46], who reported a 5.7% prevalence in sheep at the Elfora Abattoir in Bishoftu. On the other hand, the rates reported in the current study are slightly higher than the 1.3% prevalence reported for both sheep and goats in Negele town, West Guji, Ethiopia [47]. These differences in prevalence across studies may be attributed to several factors, including variations in slaughterhouse sanitation levels, hygiene practices of abattoir workers, sampling techniques, laboratory methodologies, seasonal timing of sample collection, and geographic differences [48].

In the present study, a greater prevalence of *E. coli* O157:H7 was detected in fecal samples (3.6%) than in skin swabs (2.7%) from sheep and goats. This finding is consistent with a previous report by Elder et al. [49], who reported a significantly greater prevalence in feces (28%) than in the skin (11%). However, the current results contrast with those of Abreham et al. [3], who reported a higher prevalence in skin swabs (6.9%) than in fecal samples (5.6%). These discrepancies may be due to several contributing factors. Animal skin can act as a carrier of *E. coli* O157:H7 through contact with contaminated sources such as soil, feed, water, or feces. Seasonal shedding patterns may also influence detection, with animals potentially not shedding bacteria at the time of sampling, leading to lower fecal detection rates during certain periods [50]. Furthermore, cross‐contamination of the skin may occur during transport and lairage, where animals are in close contact with one another and exposed to contaminated surfaces. This can result in higher detection rates in the skin than in the feces, particularly in environments with inadequate hygiene control.

The observed variations in the proportions of *E. coli* O157:H7 across different sample types in this study, compared with findings from other regions, can be explained by several factors. These include differences in husbandry and animal management practices, agroclimatic conditions, sampling techniques, and detection methods, as well as variations in animal breeds and ages [41, 50].

Despite immersion in hot water between trimming consecutive carcasses, *E. coli* O157:H7 was still isolated from one of the knives. This finding is consistent with previous studies by Abreham et al. [3] and Eshetu et al. [51], who reported the presence of *E. coli* O157:H7 on knives used in abattoirs. The detection of the pathogen on knives suggests that these tools may act as critical points of cross‐contamination, potentially facilitating the transfer of pathogenic bacteria from one carcass to another along the slaughter line. This highlights the inadequacy of current sanitation practices, particularly in terms of the effectiveness of knife sterilization procedures.

An earlier study in Ethiopia revealed that *E. coli* O157:H7 strains isolated from both human and animal sources exhibit antimicrobial resistance [33]. In the present study, *E. coli* O157:H7 isolates were found to be fully susceptible to gentamicin and kanamycin. This result is in agreement with the findings of Hiko et al. [33], who reported 100% susceptibility to these antibiotics in isolates from cattle, sheep, and goat meat. Similarly, Eshetu et al. [51] reported complete susceptibility in *E. coli* O157:H7 isolates from cattle. These results are further supported by those of Taye et al. [52], who reported that all *E. coli* isolates tested were susceptible to kanamycin. This susceptibility might have contributed to the effectiveness of these antimicrobial agents, primarily against Gram‐negative bacteria, such as those belonging to the family Enterobacteriaceae, to which *E. coli* O157:H7 belongs [45].

In this study, *E. coli* O157:H7 isolates presented 100% resistance to ampicillin, erythromycin, amoxicillin, and vancomycin and 92.3% resistance to chloramphenicol. This high level of resistance, particularly to commonly used antibiotics such as ampicillin and erythromycin, contrasts sharply with the findings of Hiko et al. [33], who reported 100% susceptibility of all isolates to these antibiotics. However, the results are in agreement with those of studies by Dulo et al. [32] and Eshetu et al. [51], who reported 100% resistance of *E. coli* O157:H7 isolates to erythromycin and ampicillin, respectively. The widespread resistance observed in the present study likely reflects the frequent and possibly indiscriminate use of these antimicrobials in veterinary medicine, both for therapeutic and prophylactic purposes.

Additionally, a moderate resistance level (69.2%) to tetracycline was detected, despite its widespread availability and routine use in livestock within the study area. In comparison, a study from Saudi Arabia [53] reported 100% resistance to tetracycline in *E. coli* O157:H7 isolates. This variation may be attributed to differences in resistance gene expression influenced by the evolving characteristics of the pathogen and diverse agroecological factors [54].

The findings of this study revealed that all *E. coli* O157 isolates were resistant to multiple drugs. Previous studies have reported MDR frequencies ranging from 22.6% to 100% [33, 55–57]. Such widespread MDR is likely linked to the uncontrolled or improper use of antimicrobial drugs, although this factor was not directly examined in the present study. Furthermore, the consumption of contaminated meat has been suggested as a potential pathway for the transmission of MDR bacteria in Africa [58, 59].

## 5. Conclusion

In the present study, *Escherichia coli* O157:H7 was isolated from a variety of samples collected from sheep and goats. The contamination rates were 2.88% in sheep and 2.34% in goats. All the isolates presented high levels of resistance to ampicillin, erythromycin, amoxicillin, and vancomycin but remained fully susceptible to gentamicin and kanamycin. Importantly, all *E. coli* O157:H7 isolates exhibited multidrug resistance, underscoring a significant public health concern. Owing to laboratory limitations, the study did not extend to the detection of other enterohemorrhagic *E. coli* (EHEC) serotypes or the virulence genes of *E. coli* O157:H7. Nonetheless, the findings clearly indicate that raw animal‐source meat can be contaminated with *E. coli* O157:H7, which poses a risk of infection, especially through the consumption of raw or undercooked meat or via cross‐contamination in food preparation environments. These findings highlight the need to thoroughly cooking meat and practicing strict hygiene during food handling. Future studies should prioritize the molecular characterization of isolates, including the identification of antimicrobial resistance genes and virulence factors, and should incorporate whole‐genome sequencing to better understand the genetic diversity, pathogenicity, and transmission dynamics of *E. coli* O157:H7 in food animal populations.

## Author Contributions

Aklilu Asmelash contributed to the study design, data analysis, and interpretation of the results; drafted both the initial and final versions of the manuscript; and edited the manuscript. Yusuf Abrahim participated in the study design, proposal writing, sample collection, and laboratory work. Ashenafi Syoum participated in the data analysis, drafted the initial versions of the manuscript, and edited the manuscript. Gebremedhin Gebrezgabiher participated in the data analysis, drafted both the initial and final versions of the manuscript, and edited the manuscript.

## Funding

No funding was obtained for this study.

## Ethics Statement

The protocol of the research project was reviewed and approved by Samara University, College of Veterinary Medicine, and Animal Science Ethical Review Committee.

## Conflicts of Interest

The authors declare no conflicts of interest.

## Data Availability

The data that support the findings of this study are available from the corresponding author upon reasonable request.
